# Identification of the Metabolomics Signature of Human Follicular Fluid from PCOS Women with Insulin Resistance

**DOI:** 10.1155/2022/6877541

**Published:** 2022-03-20

**Authors:** Ran Liu, Shun Bai, Shengxia Zheng, Xinyi Zhu, Yan Zhang, Bo Xu, Weidong Zhao

**Affiliations:** ^1^Cheeloo College of Medicine, Shandong University, Jinan, Shandong 250012, China; ^2^Reproductive and Genetic Hospital, The First Affiliated Hospital of USTC, Division of Life Sciences and Medicine, University of Science and Technology of China, Hefei, Anhui 230001, China

## Abstract

**Aims:**

To explore the effect of IR on the metabolism of PCOS by analyzing the changes in FF metabolites in PCOS patients who are undergoing assisted reproductive technology based on the metabonomic platform of ultraperformance gas chromatography coupled to mass spectrometry (GC/MS).

**Method:**

Eight PCOS patients with IR (PCOS-IR) and 8 PCOS patients without IR (PCOS-NIR) were enrolled. All patients received controlled ovarian stimulation by using the gonadotropin-releasing hormone (GnRH) antagonist protocol, and the FF of a single dominant follicle was collected on the day of oocyte retrieval. The metabolite profiles of the FF were determined by GC/MS. *Key Results*. A total of 20 differentially expressed metabolites in FF were identified. Compared with levels in the PCOS-NIR group, stearic acid, palmitic acid, pentadecanoic acid, stigmasterol, citric acid, isocitric acid, thymine, and pyruvic acid in FF were significantly increased in the PCOS-IR group. Lithocholic acid and sinapinic acid in FF decreased significantly. The affected metabolic pathways with potential regulatory roles were identified by KEGG annotation.

**Conclusion:**

Compared with the PCOS-NIR group, the PCOS-IR group showed more significant metabolic abnormalities. *Implications*. These results will help us to understand the pathogenesis of PCOS combined with IR and will provide new clues for studying metabolic disorders associated with PCOS, e.g., IR.

## 1. Introduction

Polycystic ovary syndrome (PCOS) is a prevalent heterogeneous disease that is an endocrine and metabolic disorder in women worldwide. Hyperandrogenism, ovulation disorder, polycystic ovaries, and insulin resistance (IR) are common clinical features of PCOS [1]. However, the etiology and pathogenesis of PCOS are still unclear. IR and the associated compensatory hyperinsulinemia are common abnormalities in PCOS. IR has been proven to be related to abnormal glucose, amino acid, and lipid metabolism [2]. IR aggravates the symptoms of PCOS and increases the risk of complications such as cardiovascular disease and type 2 diabetes in PCOS [3]. In the present study, we used the homeostatic model assessment of insulin resistance (HOMA-IR) index, which is the simplest and most effective method, to evaluate IR.

Compared with genomics and proteomics, metabonomics is more related to physiology. PCOS causes changes in pathophysiological processes and finally leads to corresponding changes in metabolites. By analyzing some metabolites and comparing them between groups, we expect to find biomarkers of PCOS-IR, which will provide a better method for disease diagnosis.

The metabolic features of the biochemical status in the circulation can be reflected in follicular fluid (FF), which is the exudate of plasma. The metabolites required for oocyte growth and development accumulate in the FF, which provides unique conditions for oocyte development [4]. Therefore, understanding the metabolic features of FF in PCOS patients with IR may help clarify the pathogenesis of PCOS-IR. We conducted FF metabolomic analysis to investigate the metabolic changes in the PCOS-IR and PCOS without IR (PCOS-NIR) groups using gas chromatography/mass spectrometry (GC/MS). We expect to identify the metabolic characteristics and biomarkers of PCOS-IR from the perspective of metabonomics.

## 2. Materials and Methods

### 2.1. Participants

The study individuals consisted of 16 patients with PCOS. These patients were divided into an IR group (8 cases, HOMA − IR ≥ 2.69) and an NIR group (8 cases, HOMA − IR < 2.69) according to the HOMA-IR. The women's PCOS diagnoses were based on the 2003 Rotterdam criteria [5]. Each patient met at least two of the following criteria: (1) oligomenorrhea and/or anovulation, (2) clinical and/or biochemical signs of hyperandrogenism, and (3) polycystic ovaries following the exclusion of congenital adrenal hyperplasia, 21-hydroxylase-deficient nonclassical adrenal hyperplasia, androgen-secreting tumor, Cushing's syndrome, and other related disorders. All patients received in vitro fertilization/intracytoplasmic sperm injection-embryo transfer (IVF/ICSI-ET) because of the couples' sterility. The existence of serious and unstable physical diseases was the exclusion criterion for both groups.

### 2.2. Samples and Clinical Data Collection

All patients were Chinese females, and FF samples were obtained from The First Affiliated Hospital of USTC. All patients received the antagonist protocol to stimulate ovulation. When at least three follicles grew to approximately 18 mm in diameter, human chorionic gonadotropin (HCG) was administered. FF was collected 34–36 hours after HCG administration under the guidance of transvaginal ultrasound. At the first puncture of each ovary, clear FF was collected from a single large follicle. Each FF sample was centrifuged at 3000 g for 10 min. Aliquots were placed in a 1.5 mL Eppendorf tube and immediately frozen in a refrigerator at −80°C until use. Serum estradiol (E2), follicle-stimulating hormone (FSH), luteinizing hormone (LH), testosterone (T), fasting blood glucose, and fasting insulin were measured on days 2–5 of the menstrual cycle of women using an automated chemiluminescence system. The women's weight and height were also measured, and their body mass index (BMI; weight (kg)/height (m^2^)) was calculated. HOMA-IR was calculated as fasting glucose (mmol/L) × fasting insulin (mIU/L)/22.5, and the critical point was 2.69 [6].

### 2.3. Sample Preparation and Derivatization

Samples stored at -80°C were thawed at room temperature. One microliter of sample was added to a 1.5 mL Eppendorf tube with 10 *μ*L of 2-chloro-l-phenylalanine (0.3 mg/mL) dissolved in methanol as an internal standard, and the tube was vortexed for 10 s. Subsequently, 1 *μ*L of an ice-cold mixture of acetonitrile-methanol (1/2, *v*/*v*) was added. After the mixtures were vortexed for 1 min, ultrasonicated at 26°C for 5 min and stored at -20°C for 10 min, the extract was centrifuged at 12,000 rpm at 4°C for 10 min. An aliquot of 1 *μ*L of the supernatant was transferred to a glass sampling vial for vacuum drying. Subsequently, 80 *μ*L of 15 mg/mL methoxylamine hydrochloride in pyridine was added. The mixture was vortexed vigorously for 2 min and incubated at 37°C for 1.5 hours. Eighty microliters of N,O-bis(trimethylsilyl)trifluoroacetamide (BSTFA) (with 1% trimethylchlorosilane (TMCS)) and 20 *μ*L of n-hexane were added to the mixture, which was vortexed vigorously for 2 min and then derivatized for 1 hour at 70°C. The samples were incubated for 30 min before GC–MS analysis.

### 2.4. GC–MS Analysis

GC–MS analysis was performed using an Agilent 7890B gas chromatography system with an Agilent 5977A MSD system (Agilent Technologies Inc., CA, USA). An ADB-5ms fused-silica capillary column (30 m × 0.25 mm i.d., 0.25 *μ*m film thickness; Agilent J & W Scientific, Folsom, CA, USA) was used to separate the derivatives. Helium was used as the carrier gas at a constant flow rate of 1 mL/min. The injector temperature was set to 260°C. A 1.0 *μ*L sample was injected in splitless mode. The initial oven temperature was 60°C, raised to 270°C at a rate of 10°C/min, raised to 305°C at a rate of 20°C/min, and finally raised to 305°C and held for 5 min. The collision energy was 70 eV. Mass spectrometric data were acquired using electron impact ionization (70 eV) in full scan mode (m/z 50–500).

### 2.5. Data Preprocessing and Statistical Analysis

Raw data from datasets, including sample information, peak names (or retention time and m/z), and peak intensities, were log10 transformed, and the resulting data were then imported into the R ropls package. The data were processed using supervised orthogonal partial least-squares-discriminant analysis (OPLS-DA) to visualize the metabolic difference between the experimental group and the control group after mean centering and unit variance scaling. Variable importance in the projection (VIP) ranks the overall contribution of each variable to the OPLS-DA model, and those variables with VIP > 1 are considered relevant for group discrimination. Default 7-round cross-validation was used in this study. Two hundred response ranking tests were performed on the OPLS-DA model, and the corresponding OPLS-DA model was established to obtain the *R*2 and *Q*2 values of the random model to measure whether the model was overfitted. Differential metabolites were obtained according to the combination of the statistically significant threshold of VIP values obtained from the OPLS-DA model and *P* values from Student's *t*-test on the normalized peak areas of different groups. Metabolites with VIP values greater than 1.0 and *P* values less than 0.05 were regarded as differential metabolites.

### 2.6. Functional Enrichment Analysis and Pathway Topology Analysis

The metabolites with significant changes between the two groups were analyzed to uncover the metabolic pathways and further biological explanation. Potential targets were selected through the *P* value of pathway enrichment analysis and the influence value of pathway topology analysis.

### 2.7. Statistical Analysis

All values are presented as the mean ± SEM (standard error of the mean), and all data were analyzed by Student's *t*-test or ANOVA. *P* < 0.05 was considered statistically significant. All statistical tests were performed using the GraphPad Prism software, version 6.0 (GraphPad Software Inc., San Diego, CA, USA).

## 3. Results

### 3.1. Clinical Characteristics

The characteristics of the patients in the PCOS-IR and PCOS-NIR groups are summarized in Table 1. We found no differences in terms of mean age (26.6 ± 4.3 years vs. 28.4 ± 3.7 years, *P* = 0.14) or basic sex hormone levels between the two groups. However, the PCOS-IR patients had a significantly higher BMI (23.1 ± 2.9 kg/m^2^ vs. 20.8 ± 1.4 kg/m^2^, *P* = 0.033) and fasting insulin levels (125.2 ± 35.4 mIU/L vs. 60.5 ± 21.2 mIU/L, *P* = 0.008) than the PCOS-NIR patients.

### 3.2. Identification of Metabolites and Data Analysis

**Figure 1:**
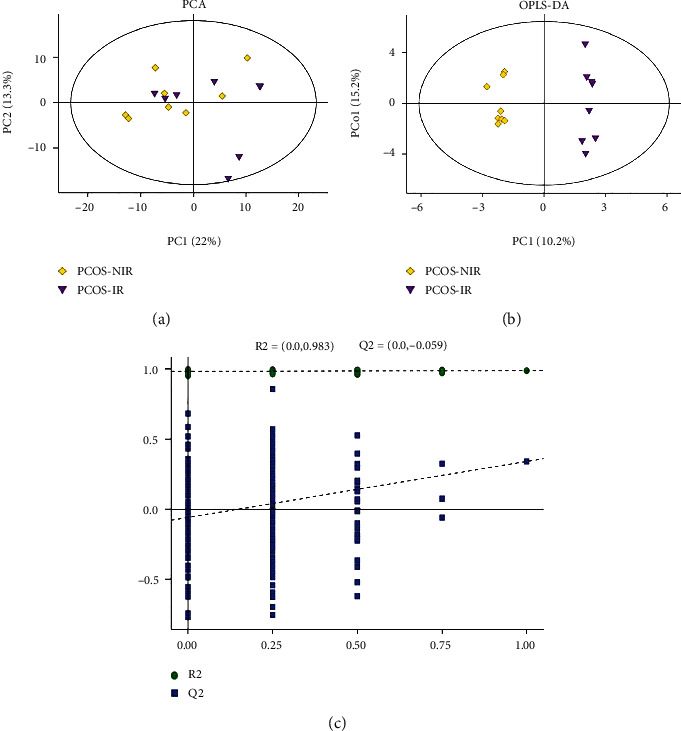
PCOS-IR patients and PCOS-NIR patients exhibited distinct metabolic characteristics. Principal component analysis (PCA) score plots were drawn according to the metabolic profiling information obtained from follicular fluid (FF) of PCOS-IR and PCOS-NIR patients (*R*2 *X* = 0.548; (a)). (b) Orthogonal projections to latent structures–discriminant analysis (OPLS-DA) score plots were drawn according to the metabolic profiling information obtained from the FF of PCOS-IR and PCOS-NIR patients (*Q*2 = 0.34, *R*2 *Y* = 0.332, and *R*2 *X* = 0.988). The purple dots indicate PCOS-IR patients (IR), and the yellow dots indicate PCOS-NIR patients (NIR). (c) The OPLS-DA model was constructed by the permutation test (*R*2 = 0.983 and *Q*2 = −0.059). The green dots indicate *R*2, and the blue dots indicate *Q*2.

**Figure 2:**
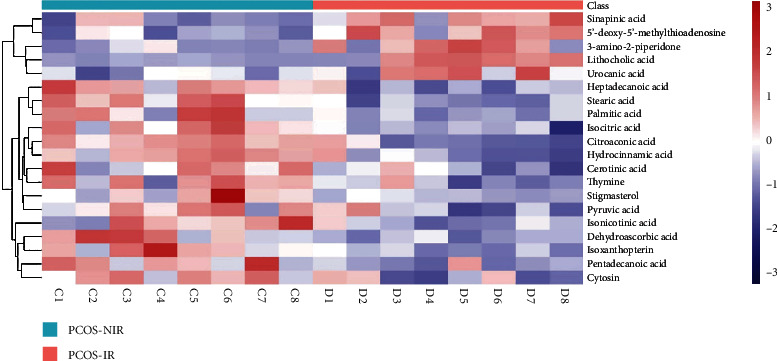
Heat map of different metabolites. The relationships between samples and the expression differences in metabolites among different samples are visually displayed. The color from blue to red indicates that the abundance of metabolites ranged from low to high. The ordinate represents the differential metabolite, and the abscissa represents the sample name. C1-C8 represent each sample of the PCOS-IR group, and D1-D8 represent each sample of the PCOS-NIR group.

**Figure 3:**
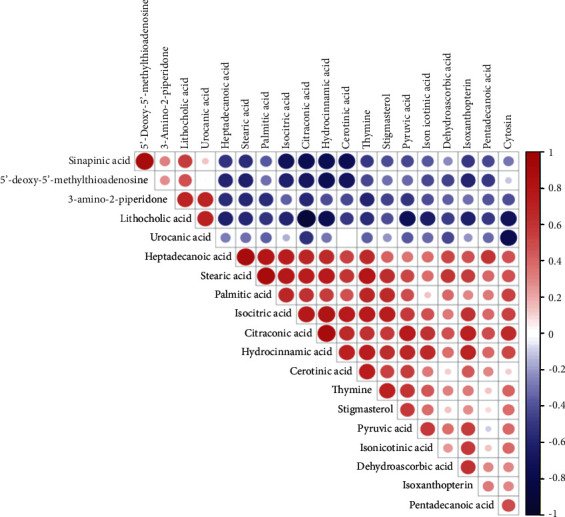
Correlation analysis of different metabolites. The Pearson correlation coefficient was used to measure the degree of linear correlation between the two metabolites; red indicates a positive correlation, and blue indicates a negative correlation.

Principal component analysis (PCA) is a type of unsuperalysis that reflects the original situation of the data (*R*2 *X* = 0.548; Figure 1(a)). Supervised OPLS-DA was used to filter out the noise irrelevant to the classification information, improve the analytical ability of the model, and highlight the differences between groups (*Q*2 = 0.34, *R*2 *Y* = 0.332, and *R*2 *X* = 0.988; Figure 1(b)). To prevent overfitting of the model, sevenfold cross-validation and 200 response permutation testing (RPT) were used to investigate the quality of the model. The supervised model was protected against overfitting by Q2 intercepting the *y*-axis at −0.059 (Figure 1(c)). Hierarchical clustering was used to analyze the expression levels of all significantly different metabolites (Figure 2). In addition, the Pearson correlation coefficient was used to measure the degree of linear correlation between the two metabolites (Figure 3). The above results show that there are significant metabolic differences between PCOS-IR and PCOS-NIR patients. In the present study, a total of 20 differential metabolites were identified (VIP > 1.0, *P* < 0.05), which are summarized in Table 2. The citric acid, isocitric acid, stearic acid, palmitic acid, pentadecanoic acid, stigmasterol, thymine, and pyruvic acid levels were significantly elevated, whereas the lithocholic acid and sinapinic acid levels were decreased in PCOS-IR patients. These changed metabolites may be related to PCOS-IR.

### 3.3. Metabolic Pathway Analysis

**Figure 4:**
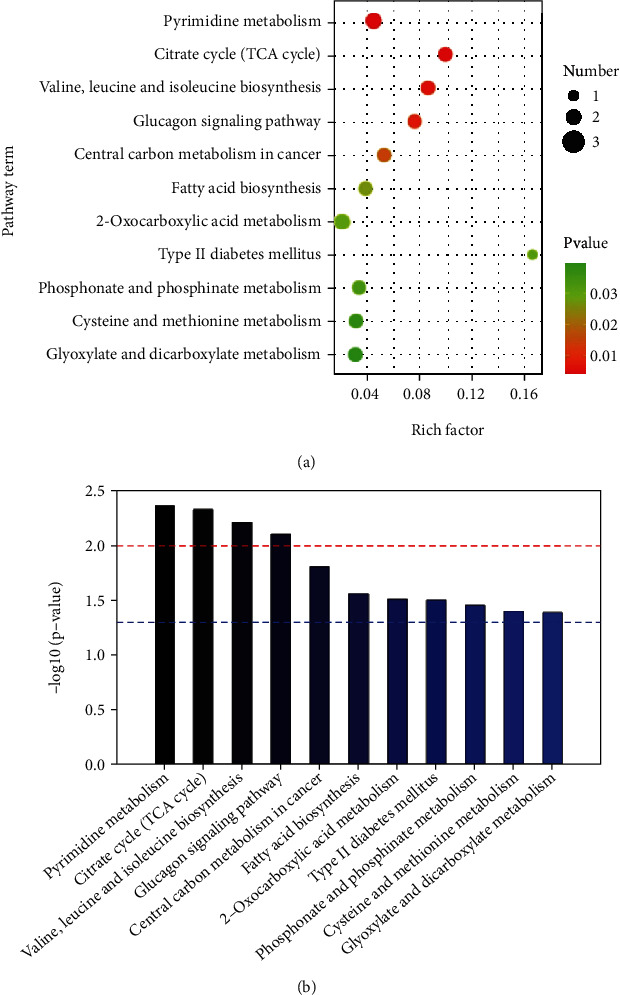
The KEGG IDs of different metabolites were used for pathway enrichment analysis to obtain the enrichment results of metabolic pathways. The significantly enriched pathways were selected for the bubble plot, and the *P* value for the metabolic pathway was the significance of the enrichment of the metabolic pathway. The ordinate is the name of the metabolic pathway, and the abscissa is the enrichment factor. The larger the rich factor is, the greater the enrichment degree is. The color from green to red indicates that the *P* value decreases; the larger the dot is, the more metabolites there were enriched in the pathway. Metabolic pathway enrichment map. The *P* value indicated by the red line is 0.01, and that indicated by the blue line is 0.05. The smaller the *P* value is, the greater the value of –log (*P* value).

**Figure 5:**
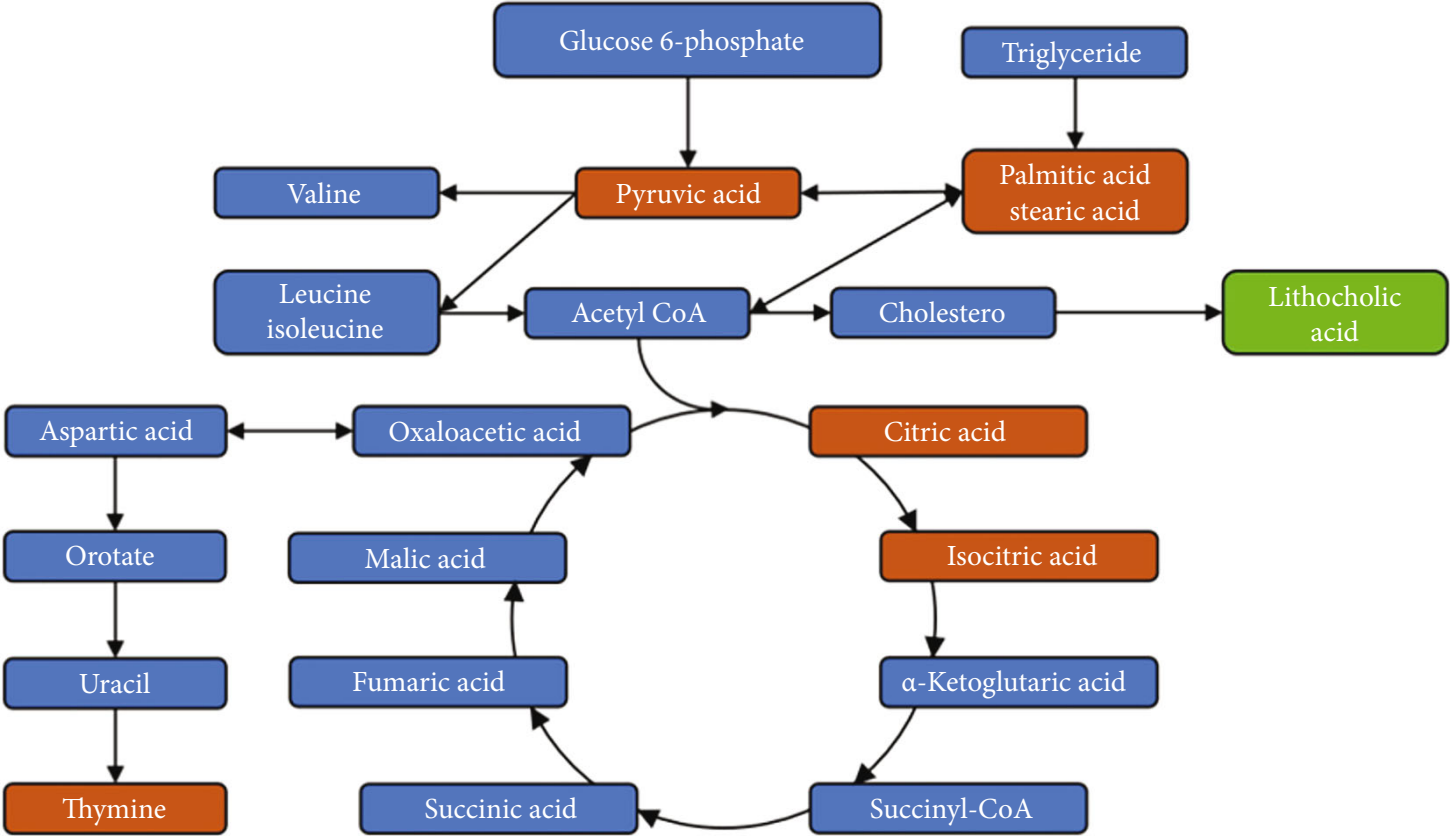
Diagram of the KEGG metabolic pathways associated with PCOS-IR development. The differential metabolites were colored according to whether they were up- or downregulated. The metabolites marked in red are those determined to be upregulated in the experiment, and the green metabolites are the downregulated metabolites. Blue color represents no significant change in metabolites.

Metabolic pathway analysis of differential metabolites is helpful for understanding the metabolic mechanisms of the differential metabolites. In this study, the metabolic pathway enrichment of differential metabolites was analyzed based on the KEGG database. The significantly enriched pathways were selected for the bubble plot (Figure 4(a)) and metabolic pathway enrichment map (Figure 4(b)). The *P* value for the metabolic pathway represents the significance of the enrichment of that metabolic pathway. *P* ≤ 0.05 was taken as the threshold. The affected metabolic pathways in PCOS-IR patients included the abnormal citrate cycle (TCA), amino acid biosynthesis, glucagon metabolic pathway, and fatty acid biosynthesis. The above results indicated that a variety of metabolic pathways were associated with PCOS-IR pathogenesis. These results confirmed the accuracy of GC–MS analysis for the detection of variations in the levels of low-concentration metabolites, which are difficult to detect with traditional methods but are often important biomarkers and indicators of metabolic defects. The differentiated metabolism in the FF of PCOS-IR patients compared with that in the PCOS-NIR patient is summarized in Figure 5, which shows the differential metabolic pathways and differential metabolites between the two groups that may be associated with PCOS-IR development. The differential metabolites were colored according to whether they were up- or downregulated. The metabolites marked in red are those determined to be upregulated in the experiment, and the green metabolites are the downregulated metabolites. Blue color represents no significant change in metabolites.

## 4. Discussion

The etiology of PCOS with IR is complex. The purpose of the present study was to investigate the metabolic characteristics of the FF of PCOS patients with IR undergoing in vitro fertilization/intracytoplasmic sperm injection-embryo transfer (IVF/ICSI-ET). The present study demonstrated the changes in metabolite profiles between PCOS-IR patients and PCOS-NIR patients, which reflected the metabolic heterogeneity among the PCOS patients. The results show that the BMI of PCOS-IR patients was significantly higher than that of the PCOS-NIR group. The levels of pyruvic acid, citric acid, isocitric acid, stearic acid, and palmitic acid were significantly higher, and the levels of lithocholic acid and sinapinic acid were significantly lower in the PCOS-IR group than in the PCOS-NIR group. In addition, the abnormal TCA cycle, amino acid biosynthesis, glucagon metabolic pathway, and fatty acid biosynthesis had significant impacts on the metabolic changes in PCOS patients with IR.

PCOS is not only a reproductive endocrine disease but also a metabolic disease that is frequently accompanied by IR and obesity [7]. The symptoms of IR are worsened with increasing BMI [8]. PCOS patients with IR have a more severe phenotype than PCOS patients without IR [7]. Niu et al. found that the level of free fatty acids (FFAs) in the PCOS group was significantly higher than that in the control group [9]. However, the previous study did not differentiate between patients with and without IR, and the samples were plasma samples from patients.

In terms of lipid metabolism, stearic acid, palmitic acid, and pentadecanoic acid were higher in patients with PCOS-IR than in patients with PCOS-NIR. Insulin can maintain the balance of lipid metabolism by inhibiting the release of FFAs from adipose tissue [10]. Specifically, inhibition of the lipid oxidation rate is weakened for patients with IR, leading to an increase in the concentration of FFAs in FF. There are dozens of FFAs in PCOS patients' plasma that are related to IR [11]. The insulin receptor can be affected by palmitic acid, which significantly stimulates insulin secretion and produces hyperinsulinemia, leading to the promotion of the occurrence of type 2 diabetes [12]. In addition, Matsuzaka et al. reported that the increase in palmitic acid and stearic acid as well as the process of converting palmitic acid to stearic acid is closely related to the occurrence of IR [13, 14]. Pentadecanoic acid may be formed from palmitic acid after intermediate hydroxylation [15]. Therefore, the increase in pentadecanoic acid in patients with PCOS-IR may be related to the increase in palmitic acid. It is speculated that the disorder of fatty acid biosynthesis in the FF of PCOS patients is closely related to IR.

Additionally, the present study showed that patients with PCOS-IR had higher levels of pyruvate, citric acid, and isocitric acid and an abnormal TCA cycle, which is the last common pathway of sugar, lipid, and amino acid metabolism. Pyruvate and isocitric acid are the main intermediate products in this cycle. Pyruvic acid, as the starting substrate for the TCA cycle and the final product of glycolysis, plays a key role in intermediary metabolism. Elevated pyruvate and fatty acid levels not only reflect the strengthening of glycolysis but also reflect the acceleration of fatty acid synthesis [16]. Pyruvate accumulation in the local tissue environment seems to be a crucial early response mechanism to hyperglycemia. Studies have found that cumulus cells in patients with PCOS show insufficiencies in the TCA cycle [17]. In addition, Rice et al. reported impaired glucose metabolism of granulosa luteal cells in PCOS patients [18]. Therefore, abnormal glucose metabolism and the TCA cycle may also be found in the FF of PCOS patients. Moreover, abnormal insulin levels in PCOS patients lead to decreased glucose uptake in the ovary and follicle [19]. More serious abnormalities in glucose metabolism and the TCA cycle may be present in patients with PCOS-IR.

Recent studies have shown that bile acid has some influence on the pathogenesis of diabetes. Bile acid metabolism is mainly mediated by bile acid nuclear receptors such as farnesoid X receptor (FXR) and membrane receptor G protein coupled receptor (TGR5), which have an effect on the secretion of endocrine factors such as insulin and glucagon [20]. Among all the classes of bile acids, lithocholic acid (LCA) is the most effective activator of TGR5. Katsuma et al. showed that TGR5 can stimulate the production of glucagon-like peptide-1 (GLP-1), which exists in intestinal endocrine cell lines and prevents IR [21]. However, insulin can inhibit the rate-limiting enzyme of bile acid synthesis, namely, 7*α* hydroxylase, thereby reducing the synthesis of bile acid. Therefore, bile acids play important roles in the control of glucose metabolism and IR. In this study, the lower level of lithocholic acid in the FF of IR patients may be related to IR. This result is consistent with previous studies [22].

The present study also showed that patients with PCOS-IR had lower levels of sinapinic acid. Sinapinic acid is a cinnamic acid derivative that has been evaluated for its potent antidiabetic activity [23]. The antihyperglycemic activity of sinapinic acid has been researched using a hyperglycemia model that was established by intraperitoneal injection of streptozotocin to destroy insulin-secreting pancreatic cells [23]. Sinapinic acid can also inhibit hydroperoxide formation more effectively by preventing lipid oxidation [24]. The hypolipidemic effect of erucic acid was also reported [25]. Therefore, the significant decrease in sinapinic acid levels in the PCOS-IR group may be related to IR and abnormal lipid metabolism.

The KEGG and pathway analysis results of this study identified differentially expressed glucagon signaling pathway and amino acid metabolic pathways in the FF of PCOS-IR patients. The physiological function of glucagon is opposite to that of insulin, and together with insulin, it regulates energy metabolism. Under physiological conditions, insulin inhibits glucagon secretion, but in the case of IR, the glucagon signaling pathway is damaged. The leucine, isoleucine, and valine biosynthesis pathways were differentially expressed between the two groups. Both valine and leucine belong to the pyruvate group of amino acids. Abnormally elevated pyruvate can potentially accelerate the biosynthesis of valine, leucine, and isoleucine [16]. To compensate for the abnormal TCA cycle, the catabolism of valine, leucine, and isoleucine may also be accelerated, which explains why there was no significant difference in amino acid metabolites between the groups. Studies have found a major impact of disordered amino acid metabolism on the development of PCOS, and the difference in the amino acid biosynthesis pathway between groups in this study may further reflect that the amino acid biosynthesis pathway is closely related to the pathogenesis of PCOS patients with IR [26]. Some studies have reported potential amino acid biomarkers for IR. Zhao et al. reported that the level of leucine was positively correlated with IR [27]. Newgard et al. proposed that the circulating concentrations of valine and leucine were positively correlated with the development of obesity-related IR [28]. Wang et al. identified valine, leucine, and isoleucine as markers of IR [29]. Wurtz et al. considered that metabolic changes in branched chain amino acids, including valine, leucine, and isoleucine, preceded hyperglycemia [30]. Therefore, the abnormal biosynthetic pathways of valine, leucine, and isoleucine may be related to the pathogenesis of IR.

## 5. Conclusion

In summary, the results of this study identified pyruvic acid, citric acid, isocitric acid, stearic acid, and palmitic acid as differential metabolites in PCOS patients with IR. Furthermore, PCOS patients with IR had an abnormal TCA cycle, amino acid biosynthesis, glucagon metabolic pathway, and fatty acid biosynthesis. Disorders of amino acid metabolism, especially valine, leucine, and isoleucine biosynthesis pathways, and the TCA cycle might play crucial roles in the development of PCOS-IR. The differences in metabolism between the two groups that we observed provided us with significant biochemical information and metabolic characteristics that enabled the diagnosis of PCOS-IR. Metabolomics will provide another perspective to help clarify the molecular mechanism underlying PCOS-IR pathogenesis.

## Figures and Tables

**Table 1:** Clinical characteristics of PCOS-IR and PCOS-NIR patients.

Clinical characteristics	Total (*n* = 16)	PCOS-IR (*n* = 8)	PCOS-NIR (*n* = 8)	*P*
Age (years), mean ± s.d.	27.5 ± 4.0	26.6 ± 4.3	28.4 ± 3.7	0.14
BMI (kg/m^2^), mean ± s.d.	22.0 ± 2.5	23.1 ± 2.9	20.8 ± 1.4	0.033
E2 (pg/mL), mean ± s.d.	45.8 ± 16.5	49.3 ± 21.4	42.4 ± 9.8	0.48
FSH (ng/mL), mean ± s.d.	6.7 ± 1.4	6.3 ± 1.8	7.0 ± 0.8	0.37
LH (ng/mL), mean ± s.d.	8.9 ± 4.4	9.2 ± 5.6	8.6 ± 3.1	0.82
LH/FSH, mean ± s.d.	1.4 ± 0.7	1.5 ± 1.0	1.2 ± 0.4	0.55
T (ng/mL), mean ± s.d.	0.7 ± 0.4	0.6 ± 0.4	0.8 ± 0.3	0.47
Fasting glucose (mmol/L), mean ± s.d.	4.8 ± 0.5	5.0 ± 0.3	4.6 ± 0.6	0.27
Fasting insulin (mIU/L), mean ± s.d.	92.8 ± 43.7	125.2 ± 35.4	60.5 ± 21.2	0.008
HOMA-IR, mean ± s.d.	2.8 ± 1.5	3.9 ± 1.2	1.8 ± 0.6	0.0098

Continuous variables are expressed as the mean ± standard deviation (SD). Student's *t*-test was used for comparisons between groups. BMI: body mass index; E2: estradiol; FSH: follicle-stimulating hormone; LH: luteinizing hormone; T: testosterone; HOMA-IR: homeostatic model index of insulin resistance.

**Table 2:** Altered metabolites between the PCOS-IR and the PCOS-NIR groups.

Metabolite name	HMDB	Class	VIP	*P* value	log2(FC)
Lithocholic acid	HMDB0000761	Lipids and lipid-like molecules	4.22019	8.41 × 10^−4^	-3.03
Citraconic acid	HMDB0000634	Fatty acyls	3.54103	1.71 × 10^−4^	1.27
Dehydroascorbic acid	HMDB0001264	Lactones	3.38468	3.55 × 10^−3^	1.57
Hydrocinnamic acid	HMDB0000764	Phenylpropanoic acids	3.19849	1.14 × 10^−3^	1.09
Isoxanthopterin	HMDB0000704	Pteridines and derivatives	2.86554	3.86 × 10^−3^	1.07
Isocitric acid	HMDB0000193	Carboxylic acids and derivatives	2.28663	1.72 × 10^−3^	0.69
3-Amino-2-piperidone	HMDB0000323	Carboxylic acids and derivatives	1.92061	5.04 × 10^−3^	-0.60
Cerotinic acid	HMDB0002356	Fatty acyls	1.78952	2.39 × 10^−2^	0.47
Urocanic acid	HMDB0000301	Azoles	1.66985	3.60 × 10^−2^	-0.77
Isonicotinic acid	HMDB0060665	Pyridines and derivatives	1.65114	1.83 × 10^−2^	0.53
Stigmasterol	HMDB0000937	Steroids and steroid derivatives	1.44124	4.80 × 10^−2^	0.42
Heptadecanoic acid	HMDB0002259	Fatty acyls	1.40184	8.77 × 10^−4^	0.24
Stearic acid	HMDB0000827	Fatty acyls	1.32349	3.51 × 10^−4^	0.21
Sinapinic acid	HMDB0032616	Cinnamic acids and derivatives	1.28220	4.96 × 10^−3^	-0.23
Pyruvic acid	HMDB0000243	Keto acids and derivatives	1.27191	4.05 × 10^−2^	0.30
Thymine	HMDB0000262	Diazines	1.24689	1.64 × 10^−2^	0.24
5′-Deoxy-5′-methylthioadenosine	HMDB0001173	5′-Deoxyribonucleosides	1.20419	1.70 × 10^−3^	-0.18
Pentadecanoic acid	HMDB0000826	Fatty acyls	1.06299	1.12 × 10^−2^	0.17
Palmitic acid	HMDB0000220	Fatty acyls	1.04755	1.15 × 10^−2^	0.17
Cytosine	HMDB0000630	Diazines	1.03637	2.10 × 10^−2^	0.19

The combination of multidimensional analysis and unidimensional analysis was used to screen the differential metabolites between the groups. VIP shows the variable weight value obtained from the OPLS-DA model with a cutoff of 1.0. The greater the VIP is, the greater the contribution of the variable to the grouping. *P* values, calculated by *t*-tests, were used to evaluate whether the differences in variables between the two groups were significant. *P* < 0.05 indicates a significant difference. The log2(FC) was calculated as the ratio of the average expression of metabolites in the two groups of samples. A positive value indicates an increase, and a negative value indicates a decrease.

## Data Availability

All raw data are available from the corresponding author upon request.

## References

[1] Bocca S., Real E. B., Lynch S. (2015). Impact of serum estradiol levels on the implantation rate of cleavage stage cryopreserved-thawed embryos transferred in programmed cycles with exogenous hormonal replacement. *Journal of Assisted Reproduction and Genetics*.

[2] Trevisan R., Nosadini R., Avogaro A. (1986). Type I diabetes is characterized by insulin resistance not only with regard to glucose, but also to lipid and amino acid metabolism. *The Journal of Clinical Endocrinology and Metabolism*.

[3] Nolan C. J., Prentki M. (2019). Insulin resistance and insulin hypersecretion in the metabolic syndrome and type 2 diabetes: time for a conceptual framework shift. *Diabetes & Vascular Disease Research*.

[4] Bayasula I., Iwase A., Kobayashi H. (2013). A proteomic analysis of human follicular fluid: comparison between fertilized oocytes and non-fertilized oocytes in the same patient. *Journal of Assisted Reproduction and Genetics*.

[5] Rotterdam EA-SPcwg (2004). Revised 2003 consensus on diagnostic criteria and long-term health risks related to polycystic ovary syndrome (PCOS). *Human Reproduction*.

[6] Yang Y., Qiao J., Li R., Li M. Z. (2011). Is interleukin-18 associated with polycystic ovary syndrome?. *Reproductive Biology and Endocrinology*.

[7] Glueck C. J., Goldenberg N. (2019). Characteristics of obesity in polycystic ovary syndrome: etiology, treatment, and genetics. *Metabolism*.

[8] Legro R. S. (2012). Obesity and PCOS: implications for diagnosis and treatment. *Seminars in Reproductive Medicine*.

[9] Dong F., Deng D., Chen H. (2015). Serum metabolomics study of polycystic ovary syndrome based on UPLC-QTOF-MS coupled with a pattern recognition approach. *Analytical and Bioanalytical Chemistry*.

[10] Teede H., Deeks A., Moran L. (2010). Polycystic ovary syndrome: a complex condition with psychological, reproductive and metabolic manifestations that impacts on health across the lifespan. *BMC Medicine*.

[11] Niu Z., Lin N., Gu R., Sun Y., Feng Y. (2014). Associations between insulin resistance, free fatty acids, and oocyte quality in polycystic ovary syndrome during in vitro fertilization. *The Journal of Clinical Endocrinology and Metabolism*.

[12] Reynoso R., Salgado L. M., Calderon V. (2003). High levels of palmitic acid lead to insulin resistance due to changes in the level of phosphorylation of the insulin receptor and insulin receptor substrate-1. *Molecular and Cellular Biochemistry*.

[13] Matsuzaka T., Shimano H., Yahagi N. (2007). Crucial role of a long-chain fatty acid elongase, Elovl6, in obesity-induced insulin resistance. *Nature Medicine*.

[14] Vessby B., Gustafsson I. B., Tengblad S., Boberg M., Andersson A. (2002). Desaturation and elongation of fatty acids and insulin action. *Annals of the New York Academy of Sciences*.

[15] Roberts L. D., Virtue S., Vidal-Puig A., Nicholls A. W., Griffin J. L. (2009). Metabolic phenotyping of a model of adipocyte differentiation. *Physiological Genomics*.

[16] McCommis K. S., Finck B. N. (2015). Mitochondrial pyruvate transport: a historical perspective and future research directions. *The Biochemical Journal*.

[17] Zhao H., Zhao Y., Li T. (2015). Metabolism alteration in follicular niche: the nexus among intermediary metabolism, mitochondrial function, and classic polycystic ovary syndrome. *Free Radical Biology & Medicine*.

[18] Rice S., Christoforidis N., Gadd C. (2005). Impaired insulin-dependent glucose metabolism in granulosa-lutein cells from anovulatory women with polycystic ovaries. *Human Reproduction*.

[19] Wallace M., Cottell E., Gibney M. J., McAuliffe F. M., Wingfield M., Brennan L. (2012). An investigation into the relationship between the metabolic profile of follicular fluid, oocyte developmental potential, and implantation outcome. *Fertility and Sterility*.

[20] Houten S. M., Watanabe M., Auwerx J. (2006). Endocrine functions of bile acids. *The EMBO Journal*.

[21] Katsuma S., Hirasawa A., Tsujimoto G. (2005). Bile acids promote glucagon-like peptide-1 secretion through TGR5 in a murine enteroendocrine cell line STC-1. *Biochemical and Biophysical Research Communications*.

[22] Legry V., Francque S., Haas J. T. (2017). Bile acid alterations are associated with insulin resistance, but not with NASH, in obese subjects. *The Journal of Clinical Endocrinology and Metabolism*.

[23] Cherng Y. G., Tsai C. C., Chung H. H., Lai Y. W., Kuo S. C., Cheng J. T. (2013). Antihyperglycemic action of sinapic acid in diabetic rats. *Journal of Agricultural and Food Chemistry*.

[24] Kikuzaki H., Hisamoto M., Hirose K., Akiyama K., Taniguchi H. (2002). Antioxidant properties of ferulic acid and its related compounds. *Journal of Agricultural and Food Chemistry*.

[25] Roy S. J., Mainzen Prince P. S. (2013). Protective effects of sinapic acid on cardiac hypertrophy, dyslipidaemia and altered electrocardiogram in isoproterenol-induced myocardial infarcted rats. *European Journal of Pharmacology*.

[26] Hou E., Zhao Y., Hang J., Qiao J. (2021). Metabolomics and correlation network analysis of follicular fluid reveals associations between l-tryptophan, l-tyrosine and polycystic ovary syndrome. *Biomedical Chromatography*.

[27] Zhao Y., Fu L., Li R. (2012). Metabolic profiles characterizing different phenotypes of polycystic ovary syndrome: plasma metabolomics analysis. *BMC Medicine*.

[28] Newgard C. B., An J., Bain J. R. (2009). A branched-chain amino acid-related metabolic signature that differentiates obese and lean humans and contributes to insulin resistance. *Cell Metabolism*.

[29] Wang T. J., Larson M. G., Vasan R. S. (2011). Metabolite profiles and the risk of developing diabetes. *Nature Medicine*.

[30] Würtz P., Tiainen M., Mäkinen V. P. (2012). Circulating metabolite predictors of glycemia in middle-aged men and women. *Diabetes Care*.

